# Health Literacy Level and Comprehension of Prescription and Nonprescription Drug Information

**DOI:** 10.3390/ijerph19116665

**Published:** 2022-05-30

**Authors:** Meehoh Kim, David Suh, Joseph A. Barone, Sun-Young Jung, Wenchen Wu, Dong-Churl Suh

**Affiliations:** 1 College of Pharmacy, Chung-Ang University, Seoul 151-756, Korea; meehohkim@gmail.com (M.K.); jsyoung@cau.ac.kr (S.-Y.J.); 2 School of Public Health, University of Michigan, Ann Arbor, MI 48109, USA; sdivad87@gmail.com; 3 EM School of Pharmacy, Rutgers—The State University of New Jersey, Piscataway, NJ 08854, USA; jbarone@pharmacy.rutgers.edu; 4 College of Pharmacy, St. John’s University, Queens, NY 11439, USA; wuw@stjohns.edu

**Keywords:** health literacy, comprehension, nonprescription, pharmaceutical labeling, pictogram

## Abstract

The aim of this study was to investigate the level of misunderstanding of medication information in Korean adults after stratifying by level of health literacy and to identify the factors influencing the misunderstanding of medication information and reading amounts of information on OTC drug labels. A cross-sectional survey was performed with 375 adult participants using the survey instrument. Multiple linear regression analyses were performed to identify factors which influence misunderstanding of medication information. Participants misunderstood 20% of words on OTC drug labels, 9% of prescription drug instructions, and 9% of pictograms. Participants on average read 59% of the overall contents of the OTC drug labels. As prescription drugs’ dosing regimens became more complicated, the level of misunderstanding instructions increased. The level of misunderstanding words on OTC drug labels significantly decreased as participants had adequate health literacy (β = −18.11, *p* < 0.001) and higher education levels (β = −6.83, *p* < 0.001), after adjusting for the study variables. The level of misunderstanding instructions for prescription drugs increased as participants became older (β = 8.81, *p* < 0.001) and had lower education levels (β = −5.05, *p* < 0.001), after adjusting for the study variables. The level of misunderstanding pictograms was similar to that of misunderstanding instructions for prescription drug labels. The amount of reading information on OTC drug labels significantly increased as respondents had adequate health literacy (β = 9.27, *p* < 0.001), were older (β = 12.49, *p* < 0.001), or had chronic diseases (β = 7.49, *p* = 0.007). Individuals’ health literacy level, reading behaviors, and complexity of medication instructions are associated with misunderstanding of medication information. Appropriate word choices in drug labels and an improved format of medication instructions could increase understanding of medication information and prevent adverse drug reactions.

## 1. Introduction

As over-the-counter (OTC) drugs are available to patients at convenience stores or non-pharmacy stores (e.g., supermarkets), patients’ ability to read and understand drug labels or medication instructions has become one of the most critical issues in the safe use of medications. However, patients are rarely well-informed about appropriate medication usage or administration instructions [1,2,3]. This lack of understanding can lead to negative health outcomes such as personal injury, preventable adverse drug reactions, increased hospitalizations due to medication errors, and increased health-related costs [4,5,6,7,8]. OTC drugs have been available for purchase at convenience stores or non-pharmacy stores for self-treatment since November 2012 in South Korea. As access to the OTC drugs has increased, the reported cases of adverse drug reactions have increased by an annual rate of 6.86% from 183,554 in 2014 to 259,089 in 2020.

Health literacy is a concept strongly associated with patients’ ability to use and understand health-related information [9]. Individuals with limited understanding and poor health literacy skills are the most vulnerable population for experiencing medication errors [10,11,12]. Therefore, it is essential to identify specific characteristics of the medication instructions that may contribute to patients’ misunderstanding of medication information and subsequent drug abuse or misuse. Different aspects of medication information affect the risk of misunderstanding medication instructions. Previous studies have identified the characteristics of both patients and drug labels that predict the risk of patients’ misunderstanding them. Growing evidence indicates that patients with low health literacy often misunderstand prescription instructions [11,13,14,15]. Patients who experience difficulties in reading and comprehending medication instructions have been concerned with their misunderstanding, such as taking the wrong dosage or taking medications at the wrong time of day [11,14]. Although reading the instructions is necessary to acquire the information in written medication instructions or drug labels, not all patients appear to read them or understand them after reading [16].

Previous studies have helped identify various factors influencing the difficulty of reading medication instruction information, which affects medication adherence. Health literacy level, health consciousness, perceived health status, number of medications, and assistance for medication adherence were mentioned as health-related factors. Moreover, sociodemographic factors such as older age and lower education level were also found to be risk factors which lead to increased misunderstanding of medication instruction information [17,18,19,20]. To our knowledge, most prior studies primarily focused on misunderstanding of prescription drug instructions. Behaviors such as different reading patterns or intentionally skipping reading, which are associated with different levels of medication information understanding, have not been examined. Thus, this study sought to examine whether health literacy level, education, age, chronic diseases, and reading patterns are potential factors in misunderstanding wording for three different kinds of medication information on OTC drug labels, prescription drug instructions, and pictograms.

The objectives of this study were (a) to investigate the level of misunderstanding of wording on OTC drug labels, prescription drug instructions, and pictograms in Korean adults after stratifying by level of health literacy, (b) to measure the amount of information read on OTC drug labels, (c) to identify the factors influencing the misunderstanding of wording on OTC drugs labels, prescription drug instructions, pictograms, and the amounts of information read on OTC drug labels.

## 2. Materials and Methods

### 2.1. Study Design and Participants

A cross-sectional survey was performed by 375 adult participants who were sampled using the proportional quota sampling method stratified by age, gender, and residence in the Seoul metropolitan area as a representation of the general Korean population. Nearly half (45%) of the 51.8 million total population of South Korea resides in the Seoul metropolitan area including Seoul and the Gyeong-gi province [21]. To determine participant eligibility, telephone screening was conducted to exclude those who had participated in other national surveys in the past six months or who had worked in the marketing, research, or healthcare industries. Eligible persons were invited to participate in this study. 

### 2.2. Survey Instrument and Data Collection

The questionnaire was developed to measure the level of misunderstanding instructions of OTC drugs and prescription drugs (the questionnaire is included in Supplementary Materials). The questionnaire consisted of ten sections: (1) questions to measure level of compliance to medication instructions, (2) questions for measurement of health literacy, (3) questions for measuring the misunderstanding of words on labels of OTC drugs which were sold at convenience stores and supermarkets, (4) questions for measuring misunderstanding prescription drug instructions, (5) questions for measuring misunderstanding instructions with pictograms, (6) questions for measuring amounts of reading information on labels of the presented OTC drugs, (7) questions for measuring the level of needing improvement in areas of medication instructions, (8) questions for respondents’ demographic information (age, gender, education level, employment status, and marital status), (9) questions for respondents’ comorbidities that persist for longer than three months (such as hypertension, cerebrovascular stroke, myocardial infarction, hyperlipidemia, osteoarthritis, rheumatoid arthritis, tuberculosis, thyroid diseases, stomach cancer, liver cancer, colorectal cancer, breast cancer, cervical cancer, lung cancer, thyroid cancer, depression, atopic dermatitis, kidney failure, hepatitis B, hepatitis C, or cirrhosis), and (10) questions measuring respondents’ health status using the SF-36 instrument.

A pilot test of the developed questionnaire was conducted with a convenience sample of 20 people. A self-administered questionnaire was conducted between October 2014–August 2020 using group survey sessions where a professional survey moderator assisted with survey completion by explaining its purpose to the respondents. The duration of a group survey session was approximately 30 min. All participants completed and signed an informed consent form before starting the survey. This study was approved by the Institutional Review Board of Chung-Ang University (approval number: 1041078–201409-HR-136-01).

### 2.3. Measurements of Health Literacy

The participants’ health literacy level was measured using the Korean version of the Rapid Estimate of Adult Literacy in Medicine (REALM). REALM tests whether a participant recognizes common words or terminologies used in a healthcare setting and is well-validated for its ability to identify patients with low health literacy [22]. The Korean version of REALM included 66 words culturally appropriately translated from the original REALM, and its administration has been modified from an in-person interview to a self-administered questionnaire [23]. The participants rated whether they knew each of the 66 words using a 4-point scale: 1 = I have not seen the word and do not understand the word, 2 = I have seen the word before but do not understand the word, 3 = I have seen the word before and understand the word a little, and 4 = I fully understand the word. The 4-point scale values were dichotomously reclassified as “do not understand the word” (initial scale value = 1 and 2) and “understand the word” (scale value = 3 and 4). The responses of understanding the word were summed to calculate a total score which ranged from 0–66. A total REALM score below 61 was used for determining inadequate health literacy [24]. The internal consistency of the REALM was tested with the present study responses (Cronbach’s α = 0.96).

Measurements for misunderstanding words on OTC drug labels were as follows: Participants were asked if they understood the meaning of 100 words found most frequently on OTC drug labels using a 4-point scale. The scale range was 1 = I do not know this word, 2 = I have seen the word before but do not know the meaning, 3 = I have seen the word before and know its meaning a little, and 4 = I know this word. The word list was selected using the following procedures: (1) extracting all words from the available 15 OTC drug labels, including those of analgesics, cold medicines, and digestive medicines; (2) counting the number of times each word appeared on the labels; and (3) selecting the 100 most frequently used words on the labels. Words were considered as misunderstood by participants when the participants’ responded scale values were 1 or 2 for the understanding of each word. The scores of misunderstanding words on OTC labels were calculated by dividing total number of misunderstood words by 100.

Measurements for misunderstanding prescription drug instructions were as follows: The understanding of instructions for the three prescribed study drugs was measured by multiple choice questions assessing whether participants accurately understood instructions about different medications listed in the three separate prescriptions. Instructions were designed to represent three levels of regimen complexity of prescription drugs, including: (a) a prescription drug for fever and aches, (b) five prescription drugs with same dose frequencies for diabetes and hypertension, and (c) five prescription drugs with different dose frequencies for asthma.

Each medication’s instructions included a list of all prescribed drugs for a patient, directions on how to take the medications, dose frequency cautions, and medication uses. Questions with four possible answer choices about medication instructions asked, for example, “According to the above medication instruction, how many times a day does the patient need to take medicine A?” and the choices were presented as (1) once, (2) twice, (3) three times, or (4) four times. The level of misunderstanding prescription drug instructions was calculated by dividing the number of incorrect answers by 16; scores were then standardized to facilitate interpretation in the scaled range of 0–100 points. The number of respondents who misunderstood prescription drug instructions was based on those respondents who answered at least one question incorrectly on interpreting instructions listed on the prescriptions.

Measurements for misunderstanding pictograms were as follows: The level of misunderstanding pictograms for taking medications was measured by questions assessing if patients could correctly understand the meaning of 12 different pictograms which were selected by a panel group consisting of professors, pharmacists, and psychologists. The pictograms were developed by the Korea Pharmaceutical Information Center to facilitate patient understanding of medication instructions by providing graphical representations of common medication dosage intervals, warnings, cautions, side effects, and drug–food interactions. Understanding the measurement for misunderstanding pictograms was tested using a multiple-choice question with four possible answer choices for each pictogram (pictograms were listed in Supplementary Materials). Participants were instructed to pick a correct instruction/description for the pictogram. The score for misunderstanding pictograms was calculated by dividing the total number of incorrect answers by 12 (the number of all pictograms). The score was then converted to a scaled range of 0–100 points with a higher score indicating higher misunderstanding.

### 2.4. Measuring Amounts of Reading Information on OTC Drug Labels 

Participants were asked how thoroughly they read the labels of the three OTC drugs (i.e., cold medicines, non-steroidal anti-inflammatory agents for children, and digestive medications). The labels of three OTC drugs were selected based on sales volume in South Korea. The information listed in six subsections of the labels, including active ingredient(s), dosage form, uses, dosage, warnings, and cautions, was presented to participants who were then asked how much information they read. An example question asked was: “How much do you read the active ingredients section of the drug label above when you buy the medicine at a convenience store?” Respondents provided answers according to a 5-point Likert scale, ranging from 1 = not reading at all to 5 = reading all contents of the section thoroughly. Three subscales of the OTC drug labels were identified according to the underlying factor structure of the six sections of OTC drug labels. The three subscales were: (a) reading about uses and dosage, (b) reading cautions and warnings, and (c) reading the active ingredients and dosage forms. A 5-point Likert scale for each subscale was converted to a 100-point scale from 0 = not reading at all to 100 = reading all contents thoroughly. The internal consistency of the scale, which was measured using Cronbach’s alpha, was 0.96.

### 2.5. Statistical Analysis

All participants were classified into adequate health literacy and inadequate health literacy based on a REALM score cutoff value of 61. The level of misunderstanding of words on OTC drug labels, prescription drugs, and pictograms, in addition to the amount of information read on drug labels, were calculated using mean with standard deviations. Since the distributions of these variables were slightly right-skewed, the median with interquartile range was also calculated. The unweighted sum of each raw scaled score was transformed to construct aggregated (summary) scores with a range of 0–100 for external comparability purposes.

A Chi-squared test or Fisher’s exact test (for cells’ expected value ≤ 5) were used to compare categorical variables between participants with adequate and inadequate health literacy, and the student’s T-test and median test were employed for continuous variables accordingly [25,26].

The validity underlying the importance of the six subsections of OTC drug labels was tested using explanatory factor analysis with varimax rotation, followed by grouping the six subsections into three subsections [27]. The number of subsections was determined based on eigen value greater than one. Cronbach’s alpha was also used to measure the internal consistency of the scale. A multiple linear regression analysis was performed to investigate the effect of predictors (i.e., health literacy level, age, education level, presence of chronic disease) on the level of misunderstanding of words on OTC drug labels, prescription drug instructions, and pictograms. The data were analyzed using the SPSS version 26.0 statistical package (IBM, Armonk, NY, USA).

## 3. Results

As presented in Table 1, the total sample of 375 participants included 54% women and had a mean age (in years) of 42.4 ± 13.2. Nearly half the sample was working full time and 71.2% had a college-level education. Compared to participants with inadequate health literacy, those with adequate health literacy had significantly higher likelihood to be employed full time, had more education, were older (43.8 vs. 39.9 years old). 

**Figure 1:**
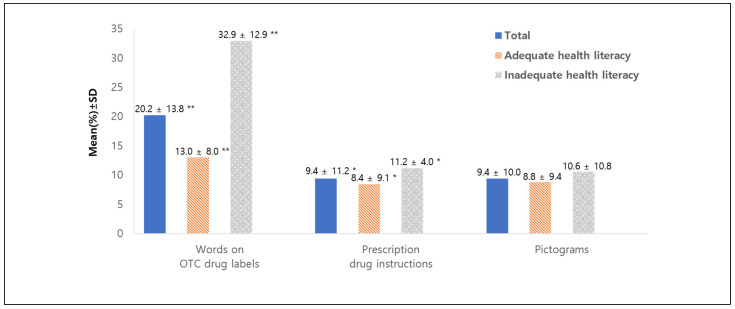
Level of misunderstanding of words on OTC drug labels, instructions for prescription drugs, and pictograms. *p*-value (* *p* < 0.05, ** *p* < 0.001) indicates statistical differences in each variable between adequate health literacy vs. inadequate health literacy groups. Abbreviations: OTC, over the counter; SD, standard deviation.

Figure 1 portrays the level of misunderstanding words on OTC drug labels, instructions for taking prescription drugs, and pictograms. Participants did not understand 20.2% of words on OTC drug labels. The words with the highest misunderstanding by participants were technical ones describing adverse drug reactions (e.g., cyanosis, kidney toxicity), drug formulations (e.g., enteric coating, sugarcoated tablet), and terminologies for certain diseases (e.g., mucocutaneous ocular syndrome, pulmonary emphysema). Participants with adequate health literacy understood statistically significantly more words than those participants with inadequate health literacy. Approximately 9% of prescription drug instructions and pictograms were misunderstood. However, the differences between participant groups were not as significant as seen in misunderstanding words of OTC drug labels.

Table 2 indicates that participants adopted selective reading patterns, such as reading an average of 59% of the overall contents of the OTC drug labels. Participants with adequate health literacy read significantly more content of the OTC drug labels (61.8%) than those with inadequate health literacy (53.4%). However, participants read much less of the cautions and warnings (53.7%) and the active ingredients and dosage sections (39.5%) compared to the information around uses and dosage instructions (84.3%). Across all information categories, those with adequate health literacy read more of the content of the OTC drug labels compared to those with inadequate health literacy (*p* < 0.001).

Table 3 portrays the proportion of participants who misunderstood prescription drug instructions depending on the therapeutic classes of medications. Among the three medications, a prescription with one medication had the lowest misunderstanding rate of 11.7%, regardless of health literacy levels. The misunderstanding rates were 19.4% for the instructions of multiple medications with the same dosage. The instructions for five medications with different dosages had the highest misunderstanding rates (21.6%). As the medication instructions became more complex, participants’ misunderstanding of them increased, particularly in participants with inadequate health literacy compared to those with adequate health literacy.

Table 4 presents the four pictograms which were most frequently misunderstood and four other pictograms which were rarely misunderstood by participants. Almost half of participants (48.8%) misunderstood the pictograms which indicated “May cause bleeding.” Conversely, the pictograms indicating “Do not take if pregnant or planning pregnancy” were correctly understood by 99% of all participants.

Table 5 presents that the magnitude of misunderstanding words on OTC drug labels was significantly decreased as participants had an adequate level of health literacy (β = 18.11, *p* < 0.001) and higher education levels (β = 6.83, *p* < 0.001) after adjusting for the study variables. The level of misunderstanding instructions for prescription drugs was increased as participants became older (β = 8.81, *p* < 0.001) and had lower education levels (β = 5.05, *p* < 0.001) after adjusting for the study variables. Lastly, the level of misunderstanding pictograms was similar to that of misunderstanding instructions for prescription drug labels. The reading amounts of information on OTC drug labels was significantly increased among participants with adequate health literacy (β = 9.27, *p* < 0.001), older age (β = 12.49, *p* < 0.001), and the presence of chronic diseases (β = 7.49, *p* = 0.007).

## 4. Discussion

The importance of research on health literacy has been steadily emerging as a topic warranting further consideration. The associations between health literacy and patterns of reading medication information or the factors influencing the misunderstanding of instructions for prescription drugs are not thoroughly understood [28]. Misunderstanding of prescription drug instructions puts individuals at risk of unintentional medication non-adherence and preventable adverse drug events [29,30]. This study has advanced the previous literature on risk factors for misunderstanding of instructions for prescription drugs by broadening the categories of medication information subject to patient interpretations, from medication instructions on OTC drug labels to pharmaceutical pictograms.

To the best of our knowledge, this study is the first to examine whether health literacy influences the ability to understand three major avenues by which medication information is given to Korean participants (e.g., the words on labels of OTC drugs; instructions for prescription drugs; and pictograms) and what factors influence patterns of reading OTC drug labels. In addition, the results of the understanding of pictograms in this study indicate the potential benefits and drawbacks of inserting them onto labels.

OTC drugs can benefit people by providing an easily accessible means of self-care if drug information can be properly understood and followed by patients without the verbal guidance of healthcare professionals. However, this study discovered that misunderstanding the wording of OTC drug labels increased as participants had inadequate health literacy and less education. This study also concluded that the amount of reading information on OTC drug label significantly increased as respondents had adequate health literacy, were older, or had chronic diseases. Participants reported not knowing the meaning of 20.2% of the most frequently found words on OTC drug labels in South Korea. Consistent with this result, prior studies on drug labels in South Korea reported that about 20% of the wording on OTC drug labels was assessed as being at a high school or above readability level, which is a level more difficult than the recommended readability levels of middle school or below [31].

Participants with inadequate health literacy significantly tended to misunderstand words on OTC drug labels compared to those with adequate health literacy. This finding is consistent with previous studies that have indicated that inadequate health literacy was associated with misunderstanding labels for prescribed medicines, although the extent to which health literacy is associated with understanding of OTC drug labels had not been examined [16]. Participants who mentioned they did not understand the meaning of the words on OTC labels reported that they also did not understand words describing adverse drug reaction symptoms [32]. Thus, patients are at greater risk for not recognizing the adverse drug reaction symptoms of OTC drugs, which may cause hospitalization and increase avoidable health care costs.

With respect to the effects of age on OTC drug label misunderstanding, this study demonstrated that misunderstanding was not influenced by age, but a previous study has indicated that misconceptions and confusion about using OTC analgesics are common among adolescents aged 14–20 years old [33]. Moreover, older adults appear to have more knowledge of and experience with using OTC drugs, but are prone to adverse drug reactions due to existing chronic health conditions and poly-pharmacy [34,35].

Many previous studies have mainly examined instructions on prescription drug labels that are typically attached to medication bottles [36,37,38]. However, a prescription is often written with multiple medications which require different dose frequencies and cautions in South Korea. Thus, this study examined the association between the level of misunderstanding and the complexity of prescription instructions.

This study’s results demonstrated that participants’ inadequate health literacy led to significantly more misunderstanding of medication instructions with low regimen complexity (e.g., one medication) compared to participants with adequate literacy but did not differentiate the rate of misunderstanding when prescription instruction complexity is moderate (e.g., multiple medications with the same dosage) or high (e.g., multiple medications with different dosages). The misunderstanding rates of instructions for prescription drugs were highest when a prescription included multiple medications with different dosages. This misunderstanding rate increased in patients with lower health literacy, older age, and lower education.

This finding suggests that healthcare professionals need to support the correct interpretation of prescriptions by providing more clarification to patients with inadequate health literacy for prescription medication instructions [39]. The results imply that the perceived difficulty of understanding medication instructions may be influenced by factors including word choice and regimen complexity. This study also revealed that misunderstanding of instructions for prescription drugs was associated with prescription characteristics, such as regimen complexity and the use of abbreviations for different dose frequencies, in addition to patient characteristics such as older age and less education. The effects of age and education on the misunderstanding of medication instructions were highly consistent with the prior studies [40,41].

The present study revealed that the understanding of pictograms was not influenced by health literacy level but understanding increased as participants were older and more educated. Pictograms for “this medicine may cause bleeding” and “take in the morning” were most often misunderstood by respondents regardless of health literacy level. In the case of the pictogram for “this medicine may cause bleeding” (which is pictured as a blood drop under a nose), a significant portion of respondents misunderstood this pictogram as “this medicine needs to be applied through the nose.” The purpose of pictogram development for assisting with medication instruction understanding for patients with inadequate literacy has been moderately achieved, as pictograms were correctly interpreted by nearly 90% of participants regardless of health literacy levels.

It is recommended that when designing pictograms, visually distinguishable shapes and colors need to be considered, and it is necessary to increase understanding by including simple explanatory text [42]. Regarding misunderstanding pictograms, previous studies have supported the results that elderly adults are particularly vulnerable to misunderstanding pictograms if the pictograms shapes are not clear or accompanied by verbal instructions [42,43,44]. However, participants’ levels of health literacy did not affect the rate of misunderstanding of pictograms.

Therefore, the current findings suggest that use of pictograms may help deliver medication instructions to less educated and older people but should be complemented with brief verbal explanations. These results strongly support a recommendation to fully utilize pictograms for medication information. Unlike labels of OTC drugs and instructions for prescription drugs, pictogram development and use does not need to be concerned with language barriers and could therefore be an effective method to train and educate patients characterized by limited education levels and older age, regardless of nationality [43].

The current findings may help healthcare professionals develop more effective strategies on communicating instructions for how to use OTC and prescription drugs and understanding pictograms according to individuals’ levels of health literacy, reading patterns, and other demographic characteristics [40]. Patients’ health literacy levels can be improved by delivering accurate information, methodical education, and effective communication [39]. Therefore, the results imply that an accurate understanding of OTC drug labels may be promoted by developing labels with a low difficulty of literacy wording for the general public in addition to educational materials, which could incorporate information on common drug formulation, therapeutic classes, and adverse drug reactions [45]. It is also necessary to develop a systematic educational program to enhance the health literacy of the general public [46].

The findings of this study should be interpreted with caution due to some limitations. First, data was collected from highly educated participants, of whom 71% graduated from college, so the study results may not be generalizable to countries in which the education level of the general population is low. Second, the present study applied a cross-sectional design, from which it is difficult to derive causal relationships between the exposure and outcomes. However, this design was used to obtain preliminary information for future cohort studies and is useful for public planning, monitoring, and evaluation [47,48]. Finally, this study data was collected using self-administered questionnaires, which might have been affected by the response bias of participants. Despite its limitations, this study has strengths in its recruitment of a representative sample of Korean adults. This current study reveals both the patient and instructional factors that may contribute to more errors in interpreting various medication information such as the OTC drug labels, prescription drug instructions, and pictograms, and the findings are consistent with those of previous studies [49,50].

## 5. Conclusions

This study revealed that lowering the reading difficulty and having clear instructions can be critical to adults who are older and have inadequate levels of health literacy and less education in improving the understanding of drug labels and prescription drug instructions, in addition to facilitating the use and correct interpretation of pictograms. Educational information leaflets that focus on skills to effectively understand and utilize OTC drug labels, prescriptions, and pictograms could be an appropriate vehicle to improve the understanding of medication information and medication adherence. Healthcare professionals, including pharmacists, should strongly consider selecting easier and more frequently used words for use on labels of OTC drugs while also providing more concise prescription instructions for complex regimens. Future research is recommended to test newly designed OTC drug labels and pictograms with vulnerable groups (e.g., older or less educated populations) to help find appropriate information channels to assist in improving the understanding of medical information.

## Figures and Tables

**Table 1:** Demographic characteristics of participants by health literacy level.

Variables	Total (*n* = 375) *n* (%)	Adequate Health Literacy (*n* = 240) *n* (%)	Inadequate Health Literacy (*n* = 135) *n* (%)	*p*-Value *
Gender				
Women	203 (54.1)	128 (53.3)	75 (55.6)	0.679
Men	172 (45.9)	112 (46.7)	60 (44.4)	
Age (Mean ± SD)	(42.4 ± 13.2)	(43.8 ± 13.7)	(39.9 ± 12.0)	0.005
20–40	185 (49.3)	117 (48.8)	68 (50.4)	0.104
41–54	113 (30.1)	80 (33.3)	33 (24.4)	
≥55	77 (20.5)	43 (17.9)	34 (25.2)	
Employment status				
Full time	160 (42.7)	116 (48.3)	44 (32.6)	<0.001
Part time	30 (8.0)	12 (5.0)	18 (13.3)	
Housewife	107 (28.5)	79 (32.9)	28 (20.7)	
Student	40 (10.7)	18 (7.5)	22 (16.3)	
Others	38 (10.1)	15 (6.3)	23 (17.0)	
Education				
<High school	63 (16.8)	25 (10.4)	38 (28.1)	<0.001
High school	45 (12.0)	19 (7.9)	26 (19.3)	
>College	267 (71.2)	196 (81.6)	71 (52.6)	
Chronic diseases				
No	268 (71.5)	177 (73.8)	91 (67.4)	0.193
Yes	107 (28.5)	63 (26.3)	44 (32.6)	

Abbreviations: SD, standard deviation. * *p*-value indicates statistical differences in variables between adequate health literacy and inadequate health literacy groups.

**Table 2:** Amounts of information read by participants on OTC drug label.

Items	Total (*n* = 375) (%)	Adequate Health Literacy (*n* = 240) (%)	Inadequate Health Literacy (*n* = 135) (%)	*p*-Value *
Overall reading amounts of information on OTC drug labels				
Mean ± SD	58.8 ± 20.8	61.8 ± 21.2	53.4 ± 19.0	<0.001
Median (IR)	58.0 (44–74)	61.5 (47–77)	52 (40–69)	<0.001
Reading amounts of information on each section of OTC drug label				
Uses and dosage				
Mean ± SD	84.3 ± 16.7	86.5 ± 15.8	80.3 ± 17.6	0.001
Median (IR)	87.5 (75–100)	91.6 (79.1–100)	83.3 (70.8–95.8)	<0.001
Cautions and warnings				
Mean ± SD	53.7 ± 25.8	56.8 ± 26.4	48.3 ± 23.7	0.002
Median (IR)	55 (35–73.3)	56.6 (38.3–76.6)	48.3 (30–66.6)	0.002
Active ingredients and dosage form				
Mean ± SD	39.5 ± 28.9	43.7 ± 29.3	31.9 ± 26.5	<0.001
Median (IR)	37.5 (12.5–62.5)	37.5 (18.7–68.7)	25 (6.2–50.0)	<0.001

Abbreviations: OTC, over the counter; SD, standard deviation; IR, interquartile range. Notes: All scores were standardized into a percentage (0–100%) to facilitate interpretation. Scores indicate the amount of information read by participants. Higher scores indicate more reading of information on OTC drug labels. * *p*-value indicates statistical differences in variables between adequate health literacy and inadequate health literacy groups.

**Table 3:** Proportion of participants misunderstanding prescription drug instructions by prescription regimen complexity.

Therapeutic Class of Medications Listed in a Prescription	Instructions for Medications Listed in a Prescription	Total (*n* = 375) *n* (%)	Adequate Health Literacy (*n* = 240) *n* (%)	Inadequate Health Literacy (*n* = 135) *n* (%)	*p*-Value *
Fever and aches	1 medication	44 (11.7)	20 (8.3)	24 (17.7)	0.001
Diabetes and hypertension	5 medications with same dosage	73 (19.4)	37 (15.4)	36 (26.6)	0.002
Asthma	5 medications with different dosages	81 (21.6)	47 (19.5)	34 (25.1)	0.060

* *p*-value indicates statistical differences in variables between adequate health literacy and inadequate health literacy groups, based on Chi-squared test.

**Table 4:** Proportion of participants misunderstanding medication pictograms.

Pictogram	Meaning	Total (*n* = 375) *n* (%)	Adequate Health Literacy (*n* = 240) *n* (%)	Inadequate Health Literacy (*n* = 135) *n* (%)
Pictograms with high misunderstanding				
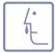	May cause bleeding	183 (48.8)	112 (46.6)	71 (52.5)
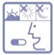	Take in the morning	46 (12.2)	33 (13.7)	13 (9.6)
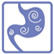	May cause stomach problems	42 (11.2)	23 (9.5)	19 (14.0)
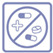	Do not take with other medicines	32 (8.5)	22 (9.1)	10 (7.4)
Pictograms with low misunderstanding				
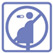	Do not take if pregnant or planning pregnancy	1 (0.2)	0 (0)	1 (0.7)
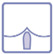	Insert into your rectum	2 (0.5)	0 (0)	2 (1.4)
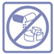	Do not break or crush	13 (3.4)	8 (3.3)	5 (3.7)
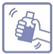	Shake it before use	14 (3.7)	7 (2.9)	7 (5.1)

**Table 5:** Factors influencing the misunderstanding and the reading amount of medication information.

Variables	Misunderstanding Words on OTC Drug Labels	Misunderstanding Prescription Drug Instructions	Misunderstanding Pictograms	Overall Reading Amount of Information on OTC Drug Labels
	Coefficient (95% CI)	Coefficient (95% CI)	Coefficient (95% CI)	Coefficient (95% CI)
Health literacy				
Inadequate	Ref	Ref	Ref	Ref
Adequate	−18.11 (−20.28, −15.95) **	−1.26 (−3.58, 1.05)	−0.26 (−2.36, 1.83)	9.27 (4.36, 14.17) **
Age				
20–40	Ref	Ref	Ref	Ref
41–54	−0.86 (−3.30, 1.57)	1.73 (−0.88, 4.34)	2.53 (0.16, 4.90) *	9.44 (3.91, 14.98) **
≥55	−1.48 (−4.59, 1.63)	8.81 (5.47, 12.14) **	5.26 (2.23, 8.28) **	12.49 (5.42, 19.55) **
Gender				
Women	Ref	Ref	Ref	Ref
Men	3.74 (1.72, 5.76) **	0.38 (−1.78, 2.54)	−0.05 (−2.01, 1.91)	−1.83 (−6.41, 2.74)
Education level				
<High school	Ref	Ref	Ref	Ref
High school	−1.53 (−5.77, 2.70)	−3.34 (−7.88, 1.19)	−1.49 (−5.61, 2.62)	3.68 (−5.93, 13.29)
>College	−6.83 (−9.92, −3.74) **	−5.05 (−8.36, −1.74) **	−5.25 (−8.25, −2.25) **	−0.57 (−7.57, 6.42)
Chronic disease				
No	Ref	Ref	Ref	Ref
Yes	0.33 (−2.06, 2.74)	−0.28 (−2.86, 2.28)	1.21 (−1.11, 3.55)	7.49 (2.04, 12.94) **
Model summary				
Adjusted R^2^	0.51	0.14	0.11	0.10
F statistics	56.834	10.137	7.692	7.562

* *p* < 0.05, ** *p* < 0.001. Abbreviations: CI, confidence interval; OTC, over the counter; Ref, reference group.

## Data Availability

The datasets used and analyzed in the study are available from the corresponding author on reasonable request.

## Supplementary Materials

The following supporting information can be downloaded at: https://www.mdpi.com/article/10.3390/ijerph19116665/s1, File S1: Questionnaire.
