# Supplementary material for: Health Literacy Level and Comprehension of Prescription and Nonprescription Drug Information
Source: Int J Environ Res Public Health. 2022 May 30;19(11):6665. doi: 10.3390/ijerph19116665 (PMC9180079; doi:10.3390/ijerph19116665)
Supplement: Supplementary file 1 [file ijerph-19-06665-s001.zip › ijerph-1728718-supplementary.pdf]

File S1. Questionnaire

As a research project conducted by the College of Pharmacy at Chung-Ang University, we are administering a survey to determine subjects' ability to understand medication information. This survey is conducted for academic purposes to improve patients' medication instructions. Thank you for participating.
**Health Status**

1. Which of the following is your smoking status?

① I have never smoked. ② I am a former smoker. ③ I am a current smoker.

2. Do you currently have diabetes or asthma?

① Yes: If you have **diabetes**, go to question **2-1**, if you have **asthma**, go to question **2-2**.

② No: Go to question 3.

2-1. How are you working to overcome diabetes? Please check all that apply.

① Medication ② Diet ③ No alcohol/smoking

④ Exercise ⑤ Regular physical examination

2-2. How are you working to overcome asthma? Please check all that apply.

⑥ Medication ⑦ Diet ⑧ No alcohol/smoking

⑨ Exercise ⑩ Regular physical examination

3. Have you been hospitalized **in the past year**?

① Yes: Go to question **3-1**.

② No: Go to question **4**.

3-1. Number of hospitalizations: ____________ times.

Average length of hospitalization: ____________ days.

Reason for hospitalization: ____.

4. Have you had any outpatient visits to the hospital **in the past year**?

① Yes: Go to question **4-1**.

② No: Go to question **5**.

4-1. Number of outpatient visits: __________ times.

Reasons for visits: ____.

5. If you are suffering from a **chronic disease**, please check (✓ or ×) the box labeled "Presence of disease" for your condition in the list below. Please check all diseases that apply to you. Also, please give the "Onset age," or the age at which your symptoms began and/or you were diagnosed. If you are currently taking any medications related to the disease, please check (✓ or ×) the "Taking medication" box.

Name of a disease	Presence of disease	Onset age	Taking medication
1) Hypertension			
2) Cerebrovascular disease (stroke)			
3) Heart disease (myocardial infarction)			
4) Dyslipidemia			
5) Osteoarthritis			
6) Rheumatoid arthritis			
7) Thyroid disease			
8) Stomach cancer			
9) Liver cancer			
10) Colon cancer			
11) Breast cancer			
12) Cervical cancer			
13) Lung cancer			
14) Thyroid cancer			
15) Depression			
16) Atopic dermatitis			
17) Kidney failure			
18) Hepatitis B			
19) Hepatitis C			
20) Cirrhosis			

6. Have you ever experienced side effects from medications you have taken in the past?

① Yes: Please select all of the following that applied to your experience by circling the symptom.

Dry mouth	Diarrhea	Constipation	Vomiting	Dizziness	Itching	Abdominal pain	Insomnia	Depression

Other: ____.

② No.

7. How did you deal with these medication side effects?

① I did not do anything
② I went to the emergency room
③ I consulted a doctor
④ I asked a pharmacist
⑤ I asked a nurse
⑥ I contacted the pharmaceutical company
⑦ I searched the Internet
⑧ I found and read the medication's packaging/inserts or attached documents

Behaviors Regarding Medication Instructions

8. When you take your medicine, do you take it as directed? Please circle one choice on the scale below.

1	2	3	4	5
**Never**	**Rarely**	**Sometimes**	**Usually**	**Always**

9. What is the main reason you did not take your medication as prescribed? Please select one option.

① My symptoms got better
② The medication did not have much effect/I did not feel different when taking it
③ Side effects appeared
④ I forgot to take my medicine
⑤ I had concerns about the drug's potential side effects
⑥ I did not know how to take the medicine
⑦ This does not apply to me; I always take my medication as prescribed.

Medication Instructions

10. Which of the following is your level of literacy? Please select one option.

① I cannot read or write.

② I can read words but cannot understand written sentences.

③ I can read simple daily texts such as invitations and business cards, but I cannot understand long or complex sentences.

④ I can understand most sentences, such as newspaper articles and advertisements that I see daily, but I do not understand complex documents.

⑤ I can understand long and difficult or complex sentences and infer their meaning.

11. How much do you read medication instructions? Please circle one option on the scale.

0%	25%	50%	75%	100%
**Don't read them**	**I read a little**	**I read half**	**I read most of them**	**I read them all**

12. How do you feel about your health? Please circle one option on the scale.

1	2	3	4	5
**Poor**	**Fair**	**Good**	**Very good**	**Excellent**

♣ Please read the pictures below and answer questions 13–15.

Label Example 1

[Efficacy·Effect] Relief of common cold symptoms (runny nose, stuffy nose, sneezing, sore throat, chills, fever, headache, joint pain and muscle pain)<br>[Usage / Dosage] Adult: take 1 tablet at a time, take 30 minutes after meals 3 times a day<br>[Efficacy·Effect] Relief of common cold symptoms (runny nose, stuffy nose, sneezing, sore throat, chills, fever, headache, joint pain and muscle pain)
[Cautions for use]<br>1. Warning<br>If you are a regular drinker of three or more drinks a day, consult your doctor or pharmacist when taking this medicine or other antipyretic analgesics. May cause liver damage.
2. The following people should not take it<br>•hypersensitivity to this drug<br>•Patients with a history of asthma while taking other antipyretic analgesics or cold medicines<br>•Patients taking antidepressants, antipsychotics, mood control, antiparkinsonian drugs, etc. or who have stopped taking them within 2 weeks<br>•Patients with genetic problems such as galactose intolerance, Lapp lactase deficiency, or glucose-galactose malabsorption
3. Do not take together with cough and sputum medicines, other cold medicines, antipyretic analgesics, sedatives, rhinitis medicines containing antihistamines, motion sickness medicines, and allergy medicines.
4. The following patients should consult with a doctor, dentist, or pharmacist before taking<br>•Those whose family members are allergic to or who have experienced drug allergies (fever, rash, asthma, itching, etc.)<br>•Patients with liver/kidney/heart disease, diabetes, high blood pressure, gastroduodenal ulcer, glaucoma, difficulty urination, weak body or high fever<br>•Patients who have gastrointestinal problems such as heartburn, stomach discomfort, stomach pain, or bleeding problems<br>•Pregnant women or women who may be pregnant or lactating<br>•Those taking diabetes, gout, arthritis drugs, anticoagulants, steroids, etc.<br>•Patients with bronchitis, emphysema, or persistent cough, etc.
5. After taking, in the following cases, stop immediately and consult a doctor, dentist, or pharmacist<br>•Rash, congestion, redness •itching •nausea •vomiting •loss of appetite •constipation •swelling •difficulty urinating •persistent or severe thirst •dizziness •shock (shortness of breath, etc.) •asthma •liver dysfunction •interstitial pneumonia •cutaneous mucosal eye syndrome •toxic epidermal necrosis dissolution •no improvement in symptoms even after taking 5 to 6 doses case, etc.
6. Avoid drinking alcohol and driving cars or machinery while taking this product.
This content is a summary of the approved information for the safe choice of consumers, so be sure to check the attached document before taking it.

13. The picture above is the back of a package of cold medicine that can be purchased at convenience stores. Look at the list below and circle the appropriate score on the scale to indicate **how much you would read each item if you were purchasing this medicine.**

Contents	Don't read (0%)	Read partially (25%)	Read half (50%)	Read mostly (75%)	Read all (100%)
1. Uses and dosage	**1**	**2**	**3**	**4**	**5**
2. Directions	**1**	**2**	**3**	**4**	**5**
3. Warnings	**1**	**2**	**3**	**4**	**5**
4. The following people should not take	**1**	**2**	**3**	**4**	**5**
5. Do not take with other medicines	**1**	**2**	**3**	**4**	**5**
6. Ask a doctor or pharmacist before use	**1**	**2**	**3**	**4**	**5**
7. Stop use and ask doctor	**1**	**2**	**3**	**4**	**5**

Label Example 2

Over-the-counter drugs<br>Read the attachment carefully before using the medicine, and keep the attachment with the medicine.
[Active ingredients] in 100mL<br>ibuprofen (KP) 2g<br>Additive (preservative):<br>methyl paraoxybenzoate(KP) 30mg<br>propyl paraoxybenzoate (KP) 20mg<br>sodium Benzoate(KP) 50mg
[Product type]<br>White or pale yellow syrupy suspension with orange odor
[Efficacy・Effect]<br>Fever and pain from a cold<br>Headache, neuralgia, muscle pain, menstrual pain, pain, toothache, arthralgia, rheumatoid pain
[Usage / Dosage]<br>Children under 15 years of age: Take the following single dose 3-4 times a day<br><One dose for children>

11-14 years old: 10-13 mL 7-10 years old: 8-10 mL<br>3-6 years old: 5-8mL 1-2 years old: 3-5mL<br>It is recommended to avoid taking it on an empty stomach, and children weighing less than 30 kg should not take more than 25 mL per day.<br>For children under 2 years of age, consult with a doctor or pharmacist before feeding<br>For other efficacy/effect, usage/dose, refer to the attached document"
[Cautions for use]<br>1. Warning<br>Taking this drug increases the risk of serious cardiovascular thrombotic reactions, myocardial infarction and stroke, which can be fatal, and serious gastrointestinal adverse reactions such as gastrointestinal bleeding, ulceration and perforation may occur.
2. The following patients should not take it<br>•Gastrointestinal ulcer or bleeding •Severe liver failure •Severe heart failure •Severe blood abnormalities •Bronchial asthma or a medical history •Severe kidney disorder •Severe hypertension •Pregnant women in the last 3 months of pregnancy •Patients with hypersensitivity to ibuprofen, aspirin or other anti-inflammatory drugs •Heart Patients with pain before and after arterial bypass surgery •Patients receiving high-dose methotrexate as chemotherapy
3. Do not exceed the recommended dose or take it together with other nonsteroidal anti-inflammatory drugs.
4. The next person should consult with a doctor, dentist, or pharmacist before taking<br>•Hepatic disorders •Bleeding tendency •Ulcerative colitis •Elderly •kidney disorders •High blood pressure •Crohn's disease •Pregnant and lactating women •Blood abnormalities •History of hypersensitivity •Systemic lupus erythematosus and mixed connective tissue disease •Hypertensive drugs, antidepressants, diuretics, anti-blood coagulants, anticancer drugs, etc. taking patients
5. After taking, in the following cases, stop immediately and consult a doctor, dentist, or pharmacist<br>•Rash • Congestion and redness •Constipation •Dizziness •Skin and mucosal eye syndrome •Nausea and vomiting •Swelling •Shock (dyspnea, etc.) •Toxic epidermal necrolysis Dissolution Anorexia •Thirst •Anemia •Pain lasting more than 5 days, 3 days Persistent fever, new symptoms, etc.
6. If you take it for a cold, take it within 5 days.
This content is a summary of the approved information for the safe choice of consumers. Be sure to check the attached document before taking it.

14. The picture above is the back of the packaging for an antipyretic (lowers fever) and painkiller. Look at the list below and circle the appropriate score on the scale to indicate **how much you would read each item if you were purchasing this medicine.**

Contents	Don't read (0%)	Read partially (25%)	Read half (50%)	Read mostly (75%)	Read all (100%)
1. Active ingredients	1	2	3	4	5
2. Product type	1	2	3	4	5
3. Uses and dosage	1	2	3	4	5
4. Directions	1	2	3	4	5
5. Warnings	1	2	3	4	5
6. The following people should not take	1	2	3	4	5
7. Do not use with another drug containing acetaminophen	1	2	3	4	5
8. Ask a doctor before use if you have liver disease	1	2	3	4	5
9. Stop use and ask doctor	1	2	3	4	5
10. Additional cautions	1	2	3	4	5
11. Other information	1	2	3	4	5

Label Example 3

[Active ingredients and its quantity]<br>Biodiastige 2000 (KPC) 50.0mg<br>Lipase (KPC) 30.0mg<br>Panprosin (KPC) 20.0mg<br>Pancellase (KPC) 30.0mg<br>Crease-PEG 80.0mg<br>Digest 100 (KPC) 10.0mg<br>Bromelain 10.0mg<br>Ursodeoxycholic acid (JP) 10.0mg<br>Simethicone Powder 70.0mg
[Product type]<br>Orange rectangular film-coated tablet
[Efficacy·Effect]

Abdominal bloating due to indigestion, loss of appetite, overeating, body weight, digestion promotion, indigestion
[Usage / Dosage]<br>Adults 1 tablet at a time, take 3 times a day after meals
[Cautions for use]<br>1. Children under 7 years of age should not take this medicine.<br>2. The following patients should consult with a doctor, dentist, or pharmacist before taking.<br>Those who have allergies to themselves or their family members<br>Pregnant women or women who may become pregnant<br>people taking other drugs<br>Those who are hypersensitive to or allergic to tartrazine<br>3. If there is no improvement in symptoms even after 2 weeks of administration, stop taking it immediately and consult a doctor, dentist, or pharmacist.
Report of side effects: Korea Pharmaceutical Safety Administration 1644-6223
[Storage method]<br>Store in airtight container, room temperature (1~30 degrees)
[Period of use] 24 months from the date of manufacture
This content is a summary of the approved information for the safe choice of consumers, so be sure to check the attached document before taking it.

15. The picture above is the back of the packaging of an antacid medicine that can be purchased at a convenience store. Look at the list below and circle the appropriate score on the scale to indicate **how much you would read each item if you were purchasing this medicine.**

**Contents**	**Don't read (0%)**	**Read partially (25%)**	**Read half (50%)**	**Read mostly (75%)**	**Read all (100%)**
1. Active ingredients	**1**	**2**	**3**	**4**	**5**
2. Product type	**1**	**2**	**3**	**4**	**5**
3. Uses and dosage	**1**	**2**	**3**	**4**	**5**
4. Directions	**1**	**2**	**3**	**4**	**5**
5. Warnings and cautions	**1**	**2**	**3**	**4**	**5**
6. Side effects	**1**	**2**	**3**	**4**	**5**
7. Other information (how to store)	**1**	**2**	**3**	**4**	**5**
8. Period of use	**1**	**2**	**3**	**4**	**5**
9. Package insert	**1**	**2**	**3**	**4**	**5**

Health Literacy

♣ Please read the instructions below carefully and answer questions 16–21.

ABC tab.	Warnings and cautions
**Ingredient:**<br>Active ingredient<br>Acetamine 400 mg (per 1 tab.)	1. Regular drinkers (3 or more drinks a day) should consult a doctor or a pharmacist before taking antipyretic analgesics. These may cause liver damage.
**Product type:**<br>White, rectangular,<br>film-coated tablet	2. Do not take if you are<br>- Hypersensitive to this drug<br>- A peptic ulcer patient<br>- A severe haptic impairment patient
**Uses:**<br>Fever, cold sores<br><br>**Dosage:**<br>Take 2 tabs. every 8 hours. Do not exceed 6 tabs. in 24 hours.	3. While you are taking this medication, do not:<br>- Exceed the recommended dose<br>- Crush, chew, or dissolve the tablet. <u>Only swallow the whole tablet</u>.
**Storage:**<br>Room temperature (1–20 degrees)	4. Please consult with your doctor if you are a<br>- Patient with hepatic impairment or a history of it<br>- Patient with a history of peptic ulcer<br>- Patient with cardiac dysfunction<br>- Pregnant/lactating woman

16. Choose the correct way to take the ABC tablet.

① Swallow as it is ② Chew it ③ Break it ④ Divide it in half

17. What is the color of this medicine?

① Yellow ② White ③ Purple ④ Green

18. If you take this medicine at 12:00 noon, when will you take it again?

① 2:00 PM ② 4:00 PM ③ 6:00 PM ④ 8:00 PM

19. How many tablets can you take per day?

① 4 ② 6 ③ 8 ④ 10

20. Which of the following patients should not take this medicine?

① Cold patient ② Hypertension patient ③ Peptic ulcer patient ④ Hyperlipidemia patient

21. If you are pregnant, what is the best thing to do before taking this medicine?

① Just take it ② Consult with family ③ Consult with doctor ④ Call 911

♣ Please read the instructions below carefully and answer questions 22–26.

Medication Instructions		
***Atenolmin Tab. 50 mg**<br>White tablet<br>Store at 1–20 degrees	Dosage: 1 tab.<br>(blood pressure lowering drug)	**Once a day** Dosing days **30**<br>If your pulse rate becomes irregular, consult a professional.<br>If you have or have a history of asthma, tell your healthcare professional
***Diclozid Tab.**<br>Orange tablet<br>Store at 1–20 degrees	Dosage: 1<br>(diuretic)	**Once a day** Dosing days **30**<br>Urine volume and frequency of urination may increase at the beginning of administration.<br>If you wake up, get up slowly. Limit sunlight exposure.
***Actos Tab. 15 mg**<br>White tablet<br>Protect from light	Dosage: 1<br>(Diabetes medication)	**Once a day** Dosing days **30**<br>Edema, headache, fatigue, and hypoglycemia may occur.<br>If you have a history of liver disease, tell your provider before taking this medication.
***Glufast Tab. 10 mg**<br>White tablet<br>Store at 1–20 degrees	Dosage: 1<br>(Diabetes medication)	**Once a day** Dosing days **30**<br>Do not take this medicine if you are eating late in the day or if you have skipped meals.<br>Take just before meals.
***Losaltan Tab.**<br>White tablet<br>Store at 1–20 degrees	Dosage: 1<br>(Depressant)	**Once a day** Dosing days **30**<br>When you wake up, sit/stand up slowly. Avoid alcohol whenever possible.<br>Do not administer to pregnant or lactating women.

22. How many pills should you take in total per day?

① 2 ② 3 ③ 4 ④ 5

23. How many orange pills should you take per day?

① 0 ② 1 ③ 2 ④ 3

24. Which medication should be stored away from light?

① Atenolmin Tab. ② Diclozid Tab. ③ Actos Tab. ④ Losaltan Tab.

25. If you are pregnant, what medication should you not take?

① Atenolmin Tab. ② Diclozid Tab. ③ Actos Tab. ④ Losaltan Tab.

26. Which of the drugs below should be taken with caution if you are going to be exposed to sunlight?

① Atenolmin Tab. ② Diclozid Tab. ③ Actos Tab. ④ Losaltan Tab.

♣ Please read the instructions below carefully and answer the questions (27–31).

**Medication instruction**		
***Cough Tab.**<br>Pale yellow tablet<br>Store at 1–20 degrees	Dosage: 1<br>(expectorant)	**3 times a day** Dosing days **7**<br>Avoid excessive drinking or smoking.<br>If you experience tension, excitement, insomnia, etc., consult a professional.
***Clarityne Tab.**<br>White tablet<br>Store at 1–20 degrees	Dosage: 1<br>(allergic disease drug)	**Once a day** Dosing days **7**<br>Do not administer to pregnant or lactating women. In case of liver disease or kidney disease, inform a specialist in advance.
***Mirinol Cap.**<br>Green/White capsule<br>Store at 1–20 degrees	Dosage: 1<br>(expectorant)	**3 times a day** Dosing days **7**<br>Drink plenty of water on a regular basis.<br>Consult your healthcare professional before using in combination with antibiotics.
***Seretide 250 Diskus**<br>Protect from light	Dosage: 1<br>(Asthma)	**Once a day** Dosing days **1**<br>Do not administer this medicine during an acute asthma attack.<br>Immediately after inhalation, rinse your mouth with water or brush your teeth.

***Singulair Tab. 10mg**<br>Yellow tablet<br>Store at 1–20 degrees	Dosage: 1<br>(Asthma /rhinitis)	**Once a day**<br>Administer regularly as prescribed.<br>Tell your healthcare professional if you have aspirin intolerance	Dosing days **35**

27. How many total drugs do you need to take **only once a day**?
① 1 ② 2 ③ 3 ④ 4

28. How many types of drugs are you taking for **7 days**?
① 1 ② 2 ③ 3 ④ 4

29. How many prescriptions do you take **on the second day** of your regimen?
① 1 ② 2 ③ 3 ④ 4

30. Which of the following medications should be washed in the mouth immediately after taking?
① Seretide 250 Diskus ② Mirinol Cap. ③ Clarityne Tab. ④ Cough Tab.

31. Which of the drugs below should be taken with caution by patients with liver disease?
① Seretide 250 Diskus ② Mirinol Cap. ③ Clarityne Tab. ④ Cough Tab.

Words in Medication Instructions

32. The following words are often used in medication instructions. Please read the list below and indicate **how well you understand each word** on the scale provided below.

Words		Don't know the word	Have heard before	Roughly know the meaning	Know the exact meaning
1	Bronchial tubes	1	2	3	4
2	Anxiety disorders	1	2	3	4
3	Flora	1	2	3	4
4	Meepul	1	2	3	4
5	Unsweetened	1	2	3	4
6	Rhinitis	1	2	3	4
7	Painkiller	1	2	3	4
8	Inflammation	1	2	3	4
9	Naengha	1	2	3	4
10	Pruritus	1	2	3	4
11	Improvement	1	2	3	4
12	Complications	1	2	3	4
13	Drowsiness	1	2	3	4
14	Secretion	1	2	3	4
15	Digestive organs	1	2	3	4
16	Humidity	1	2	3	4
17	Middle finger	1	2	3	4
18	Bleeding	1	2	3	4
19	Drinking	1	2	3	4

Words		Don’t know the word	Have heard before	Roughly know the meaning	Know the exact meaning
20	Seizure	1	2	3	4
21	Kidney	1	2	3	4
22	Heart rate	1	2	3	4
23	Decrease	1	2	3	4
24	Dongmook	1	2	3	4
25	Shading storage	1	2	3	4
26	Expectoration	1	2	3	4
27	Difficulty	1	2	3	4
28	Blood glucose	1	2	3	4
29	Fever	1	2	3	4
30	Glaucoma	1	2	3	4
31	Smoking	1	2	3	4
32	Blood vessel	1	2	3	4
33	Ingestion	1	2	3	4
34	Cough	1	2	3	4
35	Dosing period	1	2	3	4
36	Respiratory obstruction	1	2	3	4
37	Dizziness	1	2	3	4
38	Cold medicine	1	2	3	4
39	Pregnant woman	1	2	3	4
40	Anti-inflammatory drugs	1	2	3	4
41	Gaenba	1	2	3	4
42	Smooth muscle	1	2	3	4

Words		Don’t know the word	Have heard before	Roughly know the meaning	Know the exact meaning
43	Take a dose	1	2	3	4
44	Stop	1	2	3	4
45	Chest	1	2	3	4
46	Relaxation	1	2	3	4
47	Lactation	1	2	3	4
48	Caffeine	1	2	3	4
49	Room temperature	1	2	3	4
50	Molpur	1	2	3	4
51	Pain	1	2	3	4
52	Vomiting	1	2	3	4
53	Digam	1	2	3	4
54	Excessive	1	2	3	4
55	Stop	1	2	3	4
56	Oral	1	2	3	4
57	Rash	1	2	3	4
58	Antibacterial	1	2	3	4
59	Liver disease	1	2	3	4
60	Insomnia	1	2	3	4
61	Disability	1	2	3	4
62	Blood pressure	1	2	3	4
63	Infectious disease	1	2	3	4
64	Ulcer	1	2	3	4
65	Large dose	1	2	3	4

Words		Don't know the word	Have heard before	Roughly know the meaning	Know the exact meaning
66	Gastric acid secretion	1	2	3	4
67	Dilatation	1	2	3	4
68	Stomach	1	2	3	4
69	Gickon	1	2	3	4
70	Respiratory system	1	2	3	4
71	Effect	1	2	3	4
72	Cholesterol	1	2	3	4
73	Operation	1	2	3	4
74	Sultz	1	2	3	4
75	Expert	1	2	3	4
76	Diarrhea	1	2	3	4
77	Overdose	1	2	3	4
78	Difficulty	1	2	3	4
79	Antibacterial	1	2	3	4
80	Flare	1	2	3	4
81	Prescription	1	2	3	4
82	Edema	1	2	3	4
83	Symptom	1	2	3	4
84	Action	1	2	3	4
85	Nouvae	1	2	3	4
86	Moisture	1	2	3	4
87	Suppress	1	2	3	4
88	Combination	1	2	3	4

Words		Don’t know the word	Have heard before	Roughly know the meaning	Know the exact meaning
89	Spice	1	2	3	4
90	Therapy	1	2	3	4
91	Fatigue	1	2	3	4
92	Relieve	1	2	3	4
93	Flush	1	2	3	4
94	Pulse	1	2	3	4
95	Asthma	1	2	3	4
96	Insulin	1	2	3	4
97	Bokdam	1	2	3	4
98	Palpation	1	2	3	4
99	Pregnancy	1	2	3	4
100	Heart	1	2	3	4
101	Chills	1	2	3	4
102	Stop drinking	1	2	3	4
103	Empty stomach	1	2	3	4
104	Excitement	1	2	3	4
105	Hypersensitivity	1	2	3	4
106	Urine retention	1	2	3	4
107	Risk	1	2	3	4
108	Respiration	1	2	3	4
109	Medical history	1	2	3	4
110	Administration	1	2	3	4

Words on Drug Labels

33. The following words are often used on drug labels. Please read the list below and indicate **how well you understand each word** on the scale provided below.

Words		Don't know the word	Have heard before	Roughly know the meaning	Know the exact meaning
1	Addictive	1	2	3	4
2	Steroids	1	2	3	4
3	Gastroduodenal ulcer	1	2	3	4
4	Bronchitis	1	2	3	4
5	Congestion	1	2	3	4
6	Joint pain	1	2	3	4
7	Deficiency	1	2	3	4
8	Side effect	1	2	3	4
9	Phlegm	1	2	3	4
10	Suspension	1	2	3	4
11	Swelling	1	2	3	4
12	Pale yellow	1	2	3	4
13	Additive	1	2	3	4
14	Manufacturer	1	2	3	4
15	Interstitial pneumonia	1	2	3	4
16	Shock	1	2	3	4
17	Digestion promotion	1	2	3	4
18	Ulcerative	1	2	3	4

Words		Don’t know the word	Have heard before	Roughly know the meaning	Know the exact meaning
19	Heart disease	1	2	3	4
20	Enteric	1	2	3	4
21	Shortness of breath	1	2	3	4
22	Peptic ulcer	1	2	3	4
23	Onset of symptoms	1	2	3	4
24	Rhinitis medicine	1	2	3	4
25	Heart failure	1	2	3	4
26	Hematologic abnormality	1	2	3	4
27	Mucocutanealocular syndrome	1	2	3	4
28	Cardiovascular system	1	2	3	4
29	Neuralgia	1	2	3	4
30	Rheumatalgia	1	2	3	4
31	Rash	1	2	3	4
32	Sedative hypnotics	1	2	3	4
33	Airtight container	1	2	3	4
34	Headache	1	2	3	4
35	Permitted articles	1	2	3	4
36	Constitution	1	2	3	4
37	Antipyretic analgesic	1	2	3	4
38	Active ingredient	1	2	3	4
39	Antihistamines	1	2	3	4

	Words	Don't know the word	Have heard before	Roughly know the meaning	Know the exact meaning
40	Content	1	2	3	4
41	Abnormal symptoms	1	2	3	4
42	Antidepressant	1	2	3	4
43	Heart failure	1	2	3	4
44	Tight container	1	2	3	4
45	Erythema	1	2	3	4
46	Myocardial infarction	1	2	3	4
47	Intolerance	1	2	3	4
48	Rectangular	1	2	3	4
49	Tablet	1	2	3	4
50	Nephrotoxicity	1	2	3	4
51	Pulmonary emphysema	1	2	3	4
52	Toothache	1	2	3	4
53	Diuretic	1	2	3	4
54	Muscle pain	1	2	3	4
55	Sugarcoated tablet	1	2	3	4
56	Anticoagulation	1	2	3	4
57	Liver damage	1	2	3	4
58	Chronic hepatonecrosis	1	2	3	4
59	Influenza	1	2	3	4
60	Dysuria	1	2	3	4

Words		Don't know the word	Have heard before	Roughly know the meaning	Know the exact meaning
61	Dyshepatia	1	2	3	4
62	Recommended dose	1	2	3	4
63	Stroke	1	2	3	4
64	Infection	1	2	3	4
65	Lactating woman	1	2	3	4
66	Crohn's disease	1	2	3	4
67	Pneumonia	1	2	3	4
68	Hypertension	1	2	3	4
69	Allergy	1	2	3	4
70	Gout	1	2	3	4
71	Anticancer drug	1	2	3	4
72	Overeating	1	2	3	4
73	Sore throat	1	2	3	4
74	Anaphylaxis	1	2	3	4
75	First-aid	1	2	3	4
76	Tissue disorder	1	2	3	4
77	Dissolution	1	2	3	4
78	Thrombotic reaction	1	2	3	4
79	Syrup	1	2	3	4
80	Preservative	1	2	3	4
81	Formulation	1	2	3	4

Words		Don't know the word	Have heard before	Roughly know the meaning	Know the exact meaning
82	Menstrual pain	1	2	3	4
83	High fever	1	2	3	4
84	Perforation	1	2	3	4
85	Anti-Parkinson's drug	1	2	3	4
86	Anorexia	1	2	3	4
87	Kidney disorder	1	2	3	4
88	Varicella	1	2	3	4
89	Abdominal bloating	1	2	3	4
90	Stomach discomfort	1	2	3	4
91	Over-the-counter drugs	1	2	3	4
92	Antipsychotic	1	2	3	4
93	Medical history	1	2	3	4
94	Date of manufacture	1	2	3	4
95	Epidermal necrosis	1	2	3	4
96	Indigestion	1	2	3	4
97	Malabsorption	1	2	3	4
98	Long-term user	1	2	3	4
99	Cyanosis	1	2	3	4

Words on Drug Labels

34. Read the words in the list below and circle the appropriate score on the scale provided below to indicate **how well you understand each word**. Please circle only one option per word.

Words		Don't know the word	Have heard before	Roughly know the meaning	Know the exact meaning
1	Fat	1	2	3	4
2	Flu	1	2	3	4
3	Pill	1	2	3	4
4	Dose	1	2	3	4
5	Eye	1	2	3	4
6	Stress	1	2	3	4
7	Smear	1	2	3	4
8	Nerves	1	2	3	4
9	Germs	1	2	3	4
10	After meals	1	2	3	4
11	Before meals	1	2	3	4
12	Disease	1	2	3	4
13	Cancer	1	2	3	4
14	Caffeine	1	2	3	4
15	Attack	1	2	3	4
16	Kidney	1	2	3	4
17	Hormones	1	2	3	4

Words		Don’t know the word	Have heard before	Roughly know the meaning	Know the exact meaning
18	Herpes	1	2	3	4
19	Seizure	1	2	3	4
20	Bowel	1	2	3	4
21	Asthma	1	2	3	4
22	Rectal	1	2	3	4
23	Incest	1	2	3	4
24	Fatigue	1	2	3	4
25	Pelvic	1	2	3	4
26	Jaundice	1	2	3	4
27	Infection	1	2	3	4
28	Behavior	1	2	3	4
29	Prescription	1	2	3	4
30	Notify	1	2	3	4
31	Gallbladder	1	2	3	4
32	Calories	1	2	3	4
33	Depression	1	2	3	4
34	Miscarriage	1	2	3	4
35	Pregnancy	1	2	3	4
36	Arthritis	1	2	3	4
37	Nutrition	1	2	3	4
38	Menopause	1	2	3	4

Words		Don’t know the word	Have heard before	Roughly know the meaning	Know the exact meaning
39	Appendix	1	2	3	4
40	Abnormal	1	2	3	4
41	Syphilis	1	2	3	4
42	Hemorrhoids	1	2	3	4
43	Nausea	1	2	3	4
44	Directed	1	2	3	4
45	Allergic	1	2	3	4
46	Menstrual	1	2	3	4
47	Testicle	1	2	3	4
48	Colitis	1	2	3	4
49	Emergency	1	2	3	4
50	Medication	1	2	3	4
51	Occupation	1	2	3	4
52	Sexually	1	2	3	4
53	Alcoholism	1	2	3	4
54	Irritation	1	2	3	4
55	Constipation	1	2	3	4
56	Gonorrhea	1	2	3	4
57	Inflammatory	1	2	3	4
58	Diabetes	1	2	3	4
59	Hepatitis	1	2	3	4

Words		Don't know the word	Have heard before	Roughly know the meaning	Know the exact meaning
60	Antibiotics	1	2	3	4
61	Diagnosis	1	2	3	4
62	Potassium	1	2	3	4
63	Anemia	1	2	3	4
64	Obesity	1	2	3	4
65	Osteoporosis	1	2	3	4
66	Impetigo	1	2	3	4

Pictograms

♣ The following questions are about the pictograms often included in medication instructions.

35. What do you think the pictogram below means?

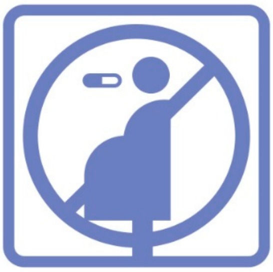

① Do not take if you are pregnant

② Women and children should not touch

③ Women should not take

④ Men should not take

36. What do you think the pictogram below means?

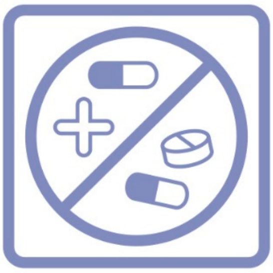

① Do not touch

② Do not take with other medicines

③ Do not eat (take)

④ Do not eat in pieces

37. What do you think the pictogram below means?

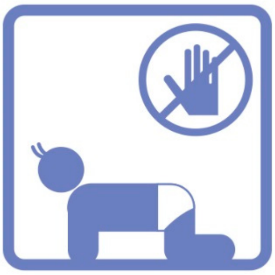

① Keep out of reach of children

② Do not touch

③ Children should not eat

④ Women should not touch

38. What do you think the pictogram below means?

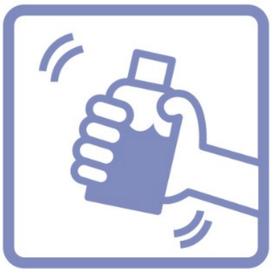

① Mouthwash

② Do not eat

③ Dissolve it in water and take it

④ Shake before use

39. What do you think the pictogram below means?

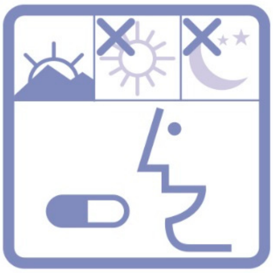

① Take it in the morning

② Take it in the afternoon

③ Take it in the evening

④ Take before bedtime

40. What do you think the pictogram below means?

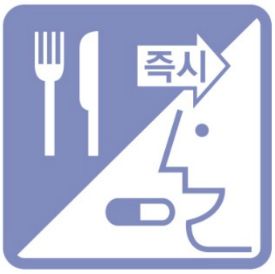

① Take immediately after meals

② Take before meals

③ Take it at the same time everyday

④ Take with meals

41. What do you think the pictogram below means?

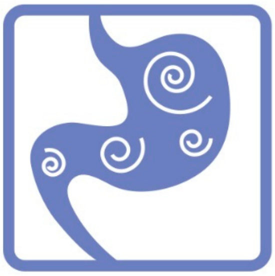

① Taking medicine

② You may feel dizzy

③ Gastrointestinal disorders may occur

④ Take on an empty stomach

42. What do you think the pictogram below mean?

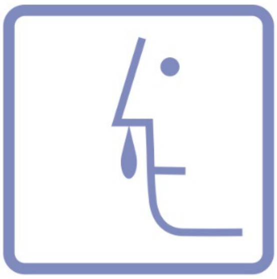

① In case of abnormal bleeding, inform a specialist

② Nose spray

③ Medicine to put in the nose

④ You may feel dizzy

43. What do you think the pictogram below mean?

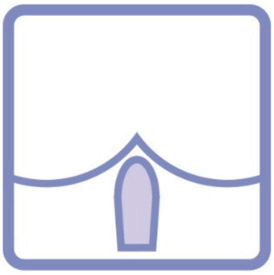

① Adhesive medicine

② Taking medicine

③ Suppository

④ Applying medicine

44. What do you think the pictogram below means?

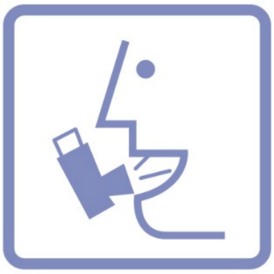

① Nose spray

② Applying medicine

③ Ear medicine

④ Inhalation

45. What do you think the pictogram below means?

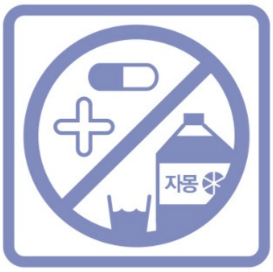

① Do not take with grapefruit juice

② Take it with grapefruit juice

③ Dissolve it in water and take it

④ Take on an empty stomach

46. What do you think the pictogram below means?

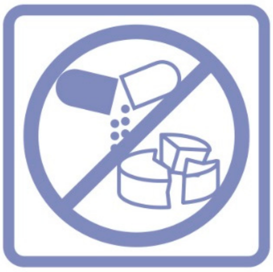

① Dissolve it in water and take it

② Shake before use

③ Do not split

④ Melt in your mouth

Demographic Questions

♣ **Please read the following questions carefully and answer.**

47. What is your gender? ________ Male ________ Female

48. What is your age? _________ years

49. What is your marital status?

① Single ② Married ③ Separated/Divorced ④ Widowed

50. Which of the following is your type of habitation?

① Single-person household ② Couple (yourself + spouse/partner
③ Yourself + Children ④ Couple + Children
⑤ Other: ____

51. Which of the following is your highest educational level?

① Dropout/graduation from elementary school ② Graduation from middle school or high school
③ Attendance at university ④ University graduation ⑤ Graduate school graduation

52. What is your average monthly income?

① < 1 million won/month ② 1–3 million won/month ③ 3–5 million won/month
④ 5–7 million won/month ⑤ 7+ million won/month

53. Which of the following is your occupational status?

① Full-time ② Part-time ③ Retired ④ Student
⑤ Laid-off or unemployed, but looking for a job ⑥ Stay-at-home spouse/partner
⑦ Other: ____

Thank you for participating.
